# Analyzing Binding Specificity in a Microparticle-Based DNA Displacement Assay Using Multiharmonic QCM-D

**DOI:** 10.1021/acs.langmuir.4c03510

**Published:** 2024-11-25

**Authors:** Taghi Moazzenzade, Luna Loohuis, Serge G. Lemay, Jurriaan Huskens

**Affiliations:** Department for Molecules and Materials, MESA+ Institute and Faculty of Science and Technology, University of Twente, P.O. Box 217, 7500 AE Enschede, The Netherlands

## Abstract

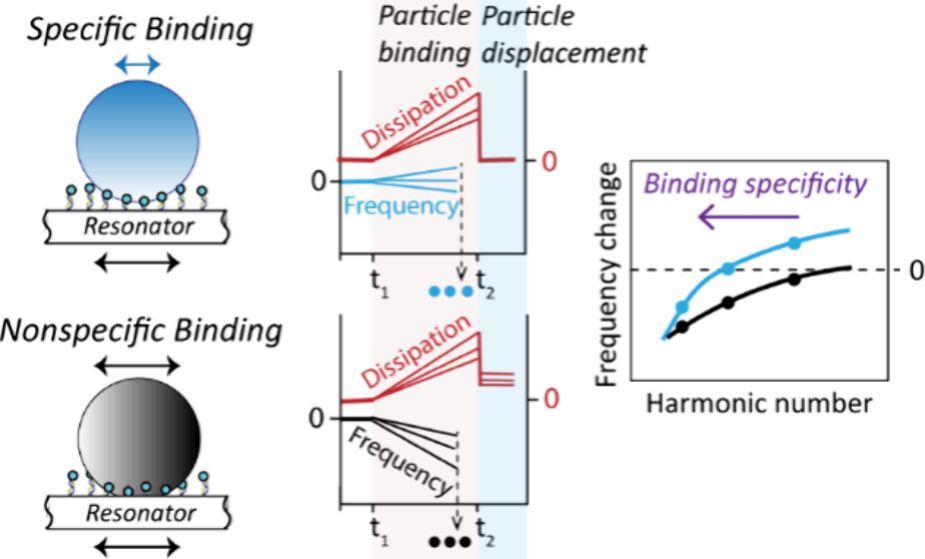

Employing particles as a label is a common approach for signal amplification in various surface-based biosensors. However, the size dependency of adhesive forces can increase the likelihood of nonspecific interactions between the particles and surface. Hence, using microscale particles in surface-based sensors requires both developing surface chemistries with enhanced antifouling properties and precise methods for evaluating these properties. Here we employ a quartz crystal microbalance with dissipation monitoring (QCM-D) to investigate the binding specificity of microparticles anchored multivalently to DNA-functionalized surfaces. We design a competitive particle displacement assay by implementing toehold-mediated strand displacement as an actuator in the microparticle–substrate interface, in which specifically anchored particles dissociate from the surface upon DNA displacement. We evaluate the efficiency of particle displacement in various modified surfaces by measuring the dissipation change (Δ*D*) following the addition of invader single-strand DNA. We further show that, prior to the particle displacement step, the specificity of particle binding can be inferred from comparing QCM-D harmonics in the particle binding step. Our results suggest that the frequency of zero crossing in the coupled-resonator model, *f*_ZC_, can be used to characterize the specificity of particle binding. In combination, *f*_ZC_ in the particle binding step and Δ*D* in the particle displacement step can be considered synergic measures for evaluating the specificity of particle binding on DNA-coated surfaces.

## Introduction

Micro- and nanoparticles can serve as signal amplification labels in surface-based biosensors.^1,2^ These amplification labels have been utilized for generating signals in electrochemical, optical, magnetic, and mass sensors for detecting various biomolecules.^3^ In particular, particle-based mechanisms have been implemented in DNA biosensors where specific hybridization of DNA biomarkers generates signals via the proximity of DNA-coated particles to the surface.^4^

Despite improvements in sensitivity, however, employing particles may undermine the specificity of biosensing by interfering with the biorecognition interaction and by increasing nonspecific interactions^5−8^ because additional adhesive forces such as van der Waals, hydrophobic, and electrostatic forces are exerted at the surface.^9^ This can become even worse when microscale particles instead of nanoscale particles are employed as the label, due to the unfavorable scaling of nonspecific adhesion forces with increasing particle size.^10−15^ Employing microparticles as the label was shown to increase nonspecific binding and generate false-positive signals.^15,16^ Additionally, the slow diffusion of microparticles can be a limiting factor in collision-based assays, such as sandwich assays. To address this, higher particle concentrations or external forces are often employed to enhance transport. However, they can also increase the likelihood of nonspecific adsorption.^17^ Hence, employing microparticles in surface-based biosensors necessitates the development of appropriate surface modifications and precise methodologies to evaluate binding specificity on modified surfaces.

Herein, we focus on evaluating the specificity of binding in a microparticle-based DNA sensor. We design a target-induced microparticle displacement assay for the detection of single-strand DNA (ssDNA) and employ multiharmonic quartz crystal microbalance with dissipation monitoring (QCM-D) responses to assess the binding specificity of microparticles on various modified surfaces. The assay is based on toehold-mediated strand displacement (TMSD):^18,19^ microparticles bound to the surface by double-strand DNA (dsDNA) can be dissociated upon displacement by a target ssDNA. Hence, this competitive assay starts with the microparticles anchored to the surface, and specifically bound particles are dissociated from the surface upon target DNA binding. We utilize the frequency of zero crossing (*f*_ZC_), together with the coupled-resonator model,^20^ to elucidate particle binding specificity as well as particle dissociation upon displacement by the target ssDNA. As a case study, we employ modified poly(l-lysine) (PLL) polymers with appended oligo(ethylene glycol) (OEG_4_) and methyltetrazine (Tz) moieties (PLL-OEG_4_-Tz)^21,22^ and hydroxy-terminated alkanethiol self-assembled monolayers (SAMs).^23^ Neutralizing the surface charge of the PLL-OEG_4_-coated surface by adding a passivant, poly(acrylic acid) (PAA), further improves the efficiency of microparticle dissociation. Our results show that for a given density of dsDNA, surfaces with a smaller *f*_ZC_ show enhanced particle dissociation, suggesting that *f*_ZC_ could serve as an indicator of particle binding specificity. This study establishes a framework for assessing the antifouling properties of surface chemistries engineered for application in microparticle-based biosensors.^15,24^

## Materials and Methods

Phosphate-buffered saline tablets (PBS, pH 7.4), poly(l-lysine)·HBr (PLL·HBr) (15–30 kDa), and 6-mercapto-1-hexanol (MCH) were purchased from Sigma-Aldrich, and Tz-OEG_4_-NHS (Tz = methyltetrazine; NHS = *N*-hydroxysuccinimide) was purchased from Click Chemistry Tools and used without further purification. Streptavidin-coated particles (0.8 μm) were purchased from Thermo-Fisher Scientific (11824992). To avoid batch-to-batch variations, for example, regarding the streptavidin density, all experiments described here were performed using a single batch of particles. Incumbent TCO-DNA (5′-TCO-PEG_4_-AAAAAAATGTGTTGATGT-3′; TCO = *trans*-cyclooctene) and the density control TCO-incumbent strand (5′-TCO-PEG4-AAAAAAAAAAAAAAAAAA-3′) were obtained from Biomers. Thiol-modified incumbent DNA (5′-SH-C6-AAAAAAATGTGTTGATGT-3′), the unmodified substrate strand (5′-CTTCCACTCACATCAACACA-3′), biotin-labeled substrate including a poly(A) sequence and a triethylene glycol spacer (5′-TEG-AAAAACTTCCACTCACATCAACACA-3′), and target DNA (5′-TGTGTTGATGTGAGTGGAAG-3′) sequences were purchased from Eurofins Genomic. Au and Pt QCM chips (AT cut, 5 MHz, 14 mm diameter) were purchased from Biolin Scientific. Milli-Q water with a resistivity of >18 MΩ cm was used in all experiments. PLL-OEG_4_(37)-Tz(1.3) with a high degree of OEG_4_ was synthesized and characterized based on previously reported works.^21,22^ QCM-D measurements were performed with a Q-Sense E4 instrument with a peristaltic pump (Biolin Scientific). All experiments were performed in a PBS buffer (pH 7.4) using a flow rate of 80 μL min^–1^. UV-ozone-treated platinum and gold substrates were used for surface functionalization. The adsorbed mass per unit area (Δ*m*) was calculated using the Sauerbrey model when calculating the density of dsDNA on the surface.

## Results and Discussion

### Design of a Competitive DNA Assay Based on TMSD

The competitive displacement assay was designed based on TMSD for the detection of a model target DNA oligonucleotide with a length of 20 nt. In TMSD, the reaction starts with a single-stranded invader (which will be the target DNA in the sensing scheme) and a primary duplex (incumbent/substrate) which possesses an overhanging single-strand domain in the substrate strand, the so-called “toehold”. The invader, which is fully complementary to the substrate, hybridizes with the toehold site of the substrate in a first-order manner, so-called toehold hybridization, and displaces the single-strand incumbent in an unbiased random walk process named branch migration.^18,25^ Because the invader is fully complementary to the substrate, the reaction is thermodynamically driven forward and leads to the displacement of the single-strand incumbent.^18^

**Figure 1:**
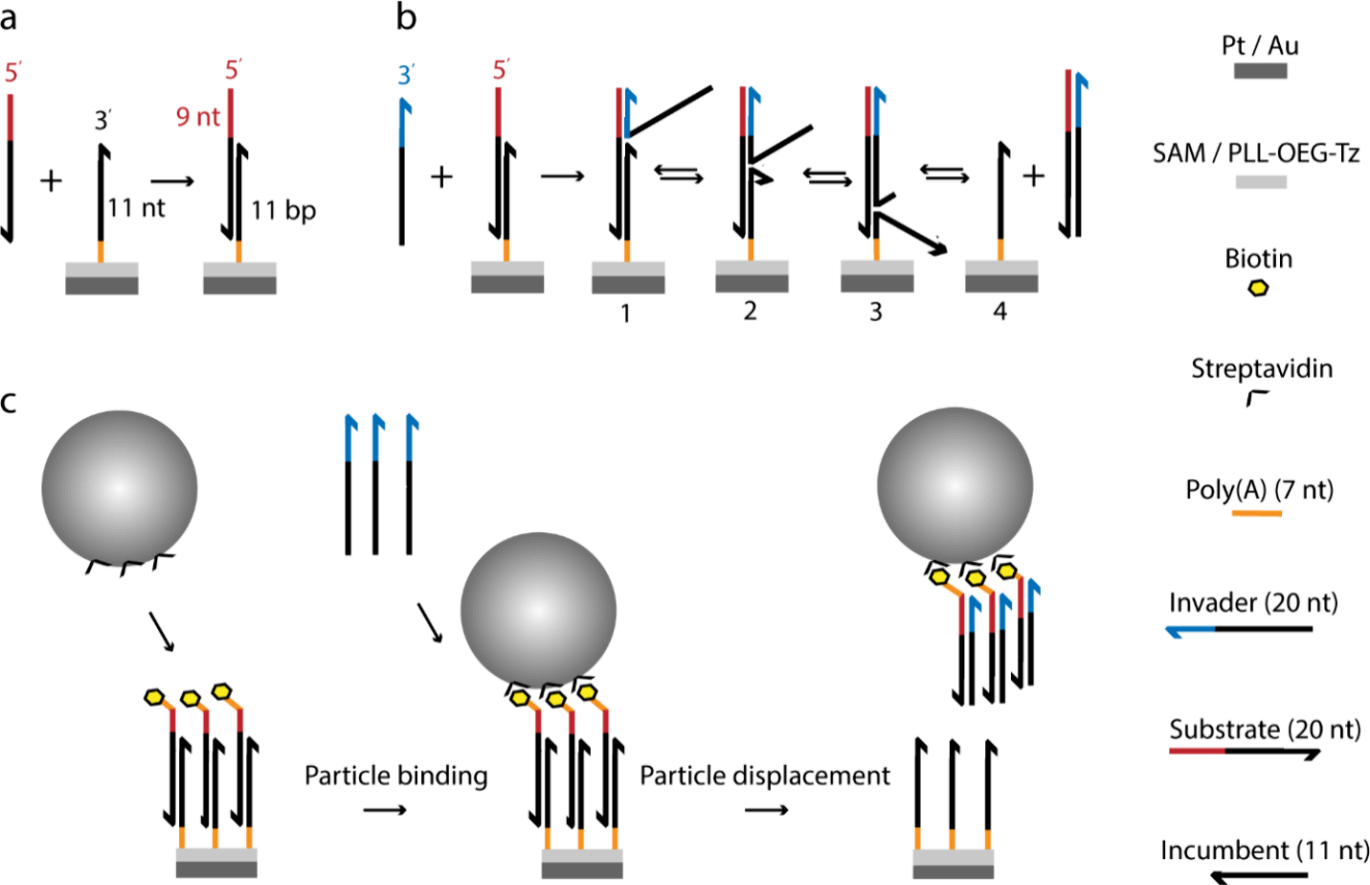
Surface-immobilized DNA displacement and particle dissociation assay. (a) Hybridization of the substrate strand on a surface with a preimmobilized incumbent. The surface-immobilized incumbent strand can hybridize with the substrate strand to form a primary duplex with a single-strand toehold at the 5′ end. (b) DNA displacement: the invader hybridizes with the toehold in the primary duplex and branch migration proceeds in a random walk process, eventually leading to the irreversible displacement of the immobilized incumbent. (c) Binding of particles on the dsDNA-coated surface and dissociation of the specifically anchored particles (800 nm) upon DNA displacement. Microparticles anchored to the DNA-coated surface through one or multiple strands dissociate upon displacement of the bound DNA strands.

Figure 1 illustrates the DNA displacement assay. The 5′-modified incumbent strand on the surface is used for the hybridization of the substrate strand (20 nt), which, in turn, functions as the probe sequence for the target DNA. This geometry results in a toehold site at the 5′ end of the hybridized substrate which is available for starting the displacement of the incumbent by the invader (i.e., the target). Due to the acceleration of the reaction kinetics with toehold length,^25,26^ a sufficiently long domain (9 nucleotides) was adopted for the toehold site. This renders the overall reaction rate insensitive to the toehold hybridization step.^18,25^ In addition, the composition and length of the incumbent/substrate duplex (11 nucleotides) were designed using the OligoAnalyzer tool to possess the minimum possible hairpin and self-dimer structures on the surface in the case of high probe densities. The surface-immobilized incumbent strand (length 11 nt) undergoes hybridization with the substrate strand (20 nt) to form a primary duplex (11 bp) together with a toehold sequence (9 nt) at the 5′ end (Figure 1a). The DNA displacement process (Figure 1b) starts with the toehold hybridization (step 1). Branch migration proceeds in a random walk process (steps 2–4). Ultimately the invader displaces the incumbent and releases the secondary duplex (invader/substrate, length = 20 bp), leaving the immobilized incumbent strand on the surface (step 5).

QCM-D was employed for analyzing the displacement process using gold-coated QCM chips as sensors. QCM is a surface-sensitive real-time sensor in which deposition or desorption of a mass can affect the resonance frequency (Δ*f*) of a driven oscillating piezoelectric crystal. In addition to the frequency shift (Δ*f*), monitoring dissipation changes (Δ*D*) enables analysis of energy loss upon deposition of viscoelastic layers and microscale objects. Here, we employ multiharmonic Δ*D* and Δ*f* responses for analyzing DNA and particle displacement assays.

**Figure 2:**
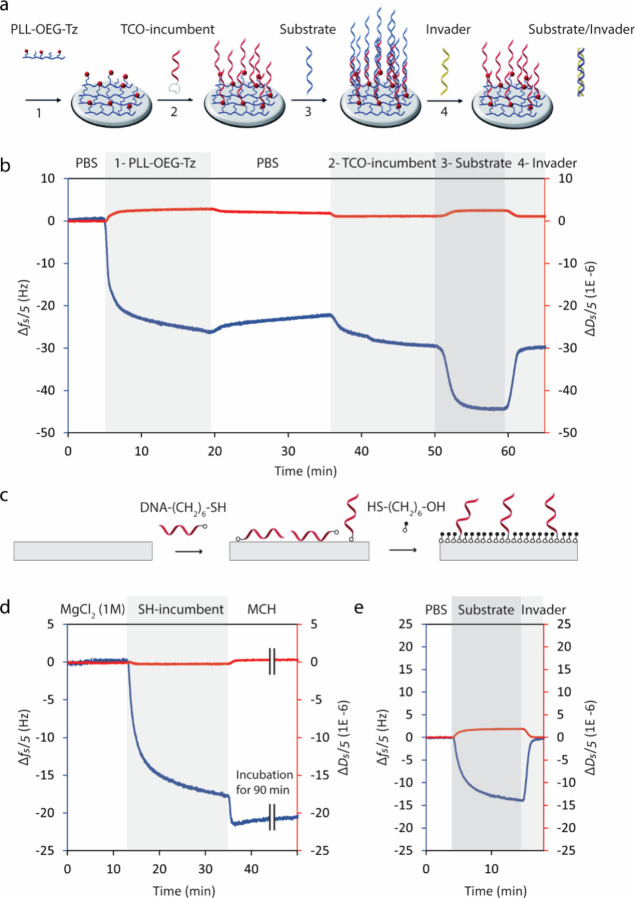
(a) Schematic representation of DNA immobilization, hybridization, and displacement on the PLL-OEG_4_-Tz-coated surface. The scheme is adapted from ref (22) with permission from the Royal Society of Chemistry. (b) Frequency and dissipation QCM-D time traces during coating of PLL-OEG_4_-Tz (1), immobilization of the incumbent TCO-DNA (2), hybridization of the substrate ssDNA (3), and displacement of the incumbent strand upon addition of invader ssDNA strand (4). (c) Schematic representation of immobilizing the thiol-modified incumbent on a Au surface followed by backfilling the surface with a hydroxy-terminated thiol. The surface modification with thiol chemistry was performed in 1 M MgCl_2_.^27^ To compare with the PLL-coated surface, the DNA hybridization and displacement measurements were performed in PBS buffer. (d) Frequency and dissipation time traces of immobilizing thiol-DNA in 1 M MgCl_2_ followed by coating the surface with hydroxy-terminated SAM. (e) Hybridization of the substrate ssDNA on the surface in part d, and displacement of the incumbent strand upon the addition of invader ssDNA strand in PBS solution. The frequency shifts for the fifth harmonic (Δ*f*_5_/5) are represented by the blue line and the dissipation changes (Δ*D*_5_/5) by the red line. The DNA displacement measurements were performed in PBS buffer, pH 7.4, and the concentration of all DNA strands was 1 μM.

The incumbent strand needs to be immobilized using a surface chemistry method that at the same time controls the probe density and the antifouling properties of the layer. Tetrazine (Tz)-modified PLL (PLL-OEG_4_-Tz), which allows reaction with *trans*-cyclooctene (TCO)-modified probes using the common strain-promoted click chemistry,^21,22^ was used to fulfill these requirements. Here, PLL-OEG_4_-Tz with a high degree of OEG_4_, 35%, was synthesized based on a previous report,^22^ and used for immobilizing the incumbent DNA. In this approach, the polymer adsorbs electrostatically on the surface via its free amine groups, OEG_4_ moieties play the role of antifouling agent, and the functional groups (Tz) undergo reaction with TCO-modified incumbent DNA. Figure 2a shows schematic representations for the DNA immobilization, hybridization, and displacement on the PLL-OEG_4_-Tz-coated surface.

Figure 2b shows the QCM-D time traces recorded while coating the surface with PLL-OEG_4_-Tz, followed by the DNA displacement assay. The negative frequency shift (step 1) reflects the mass increase upon coating the QCM substrate with the PLL polymer, caused by electrostatic adsorption of the polymer onto the activated surface to form a rigid subnanometer layer.^21^ The negligible dissipation change (Δ*D*) during the measurement confirms the small thickness and rigid nature of the adsorbed layer.^21^ The PLL-coated surface was washed with PBS buffer to remove loosely bound polymers from the surface (step 2), and then flushed with 1 μM incumbent TCO-DNA solution (step 3) for the immobilization of the TCO-modified incumbent strand. Because the dissipation change is small, we can quantify the deposited mass and the DNA density on the surface using the Sauerbrey equation; Δ*m* = −*C*Δ*f*/*n*. Here Δ*m* is the adsorbed mass per unit area (in ng cm^–2^), *n* is the overtone number, and *C* is the constant of value 17.7 ng cm^–2^ Hz^–1^ at *f* = 5 MHz for a AT cut QCM chip with a diameter of 14 mm.^28^ Using this equation, the mass per unit area can be obtained as 177 ng cm^–2^ for Δ*f*/*n* = 10 Hz. Considering the hydration mass of ≈80%, this value corresponds to the average density of ≈3.5 ssDNA molecules per (10 nm)^2^ on the surface for the incumbent DNA strand with a molecular weight of MW = 6150 g mol^–1^.^22^

Step 4 shows the hybridization of the substrate strands, which leads to a negative frequency change together with a small, anticorrelated change in dissipation. Upon the addition of the invader strand, on the other hand, the frequency exhibits a positive shift and the dissipation change becomes negligible, indicating a decreased mass upon DNA displacement (step 4). This process, as shown schematically in Figure 2a, releases the substrate/invader duplex into the solution and leads to an immobilized single-strand incumbent on the surface. Because the magnitudes of Δ*f* are comparable in the hybridization (step 3) and displacement (step 4) steps, it can be inferred that introducing the invader strand displaces the hybridized substrates completely. This result represents the dynamicity, reversibility, and accessibility of the designed DNA construct. In addition, although it might be expected that the positive charges of the free amine groups could cause nonspecific binding of negatively charged (DNA) molecules, the complete restoration of Δ*f* indicates the specificity of substrate binding of the PLL-modified surface and the ideal antifouling property of the PLL-OEG_4_-Tz chemistry for reversibly binding relatively short DNA chains. Hence, the previously reported density of OEG_4_ moieties^21^ can properly prevent nonspecific adsorption of biomolecules even when the receptor and target are highly negatively charged biomolecules such as DNA.

In a comparison of the hybridization and displacement steps in Figure 2b (steps 3 and 4, respectively), the steeper slope in the displacement step indicates that, for this actuator, DNA displacement is faster than DNA hybridization. This is even though the concentration of the substrate DNA (1 μM) is not the limiting factor in the hybridization step.^29^ The faster displacement can be due to the fast hybridization of the toehold sequence at a high concentration of invader (1 μM) and the following fast branch migration of a short sequence (11 nt), which is independent of the concentration. The displacement step is fast enough to prevent the appearance of a negative frequency shift during the toehold hybridization and branch migration steps (steps 1–4 in Figure 1), during which three DNA strands are anchored on the surface and branch migration appears as a single step in QCM measurement (step 4 in Figure 2b).

The same assay was performed using thiol chemistry, where MCH was used as the surface-passivating agent for the immobilization of thiol-modified incumbent DNA to test the strand displacement process (Figure 2c–e). As can be seen in Figure 2e, hybridization of the substrate strand and displacement of the incumbent in the hydroxy-terminated surface show behavior similar to that of the PLL chemistry, confirming the dynamicity of the actuator. The high similarity and complete displacement show the applicability of both surface chemistries for this displacement assay.

### Implementation of Strand Displacement in a Particle Dissociation Assay

The particle competitive displacement assay (Figure 1c) was performed by employing the DNA displacement construct between the particle and surface. In this assay, a biotin-labeled substrate DNA was used for the specific binding of streptavidin-coated polystyrene particles (SAv-PS, diameter 800 nm, ζ potential −30 mV). With this architecture, specifically anchored particles on a layer of dsDNA bridges can be dissociated from the surface by adding the invader (target DNA) and displacing the incumbent. On the basis of the density of dsDNA on the surface [4 dsDNA per (10 nm)^2^] and the contour length^30^ (≈3.5 nm for the 11-bp primary duplex) and also considering a high density of streptavidin proteins on the particle surface [1 protein per (10 nm)^2^], we estimate that about ≈90 dsDNA bridges can form between the particle and surface (Figure S1). However, the estimated number of contacts could be more because an immobilized streptavidin has a residual valency of two and can bind to one or two available biotin-labeled dsDNA.^31,32^ On the other hand, it is expected that not all available sites can bind simultaneously due to steric hindrance. Similar to the DNA displacement assay, this step is also expected to show complete restoration of Δ*f* and Δ*D* after the invader DNA is added to a surface, assuming an ideal antifouling property.

**Figure 3:**
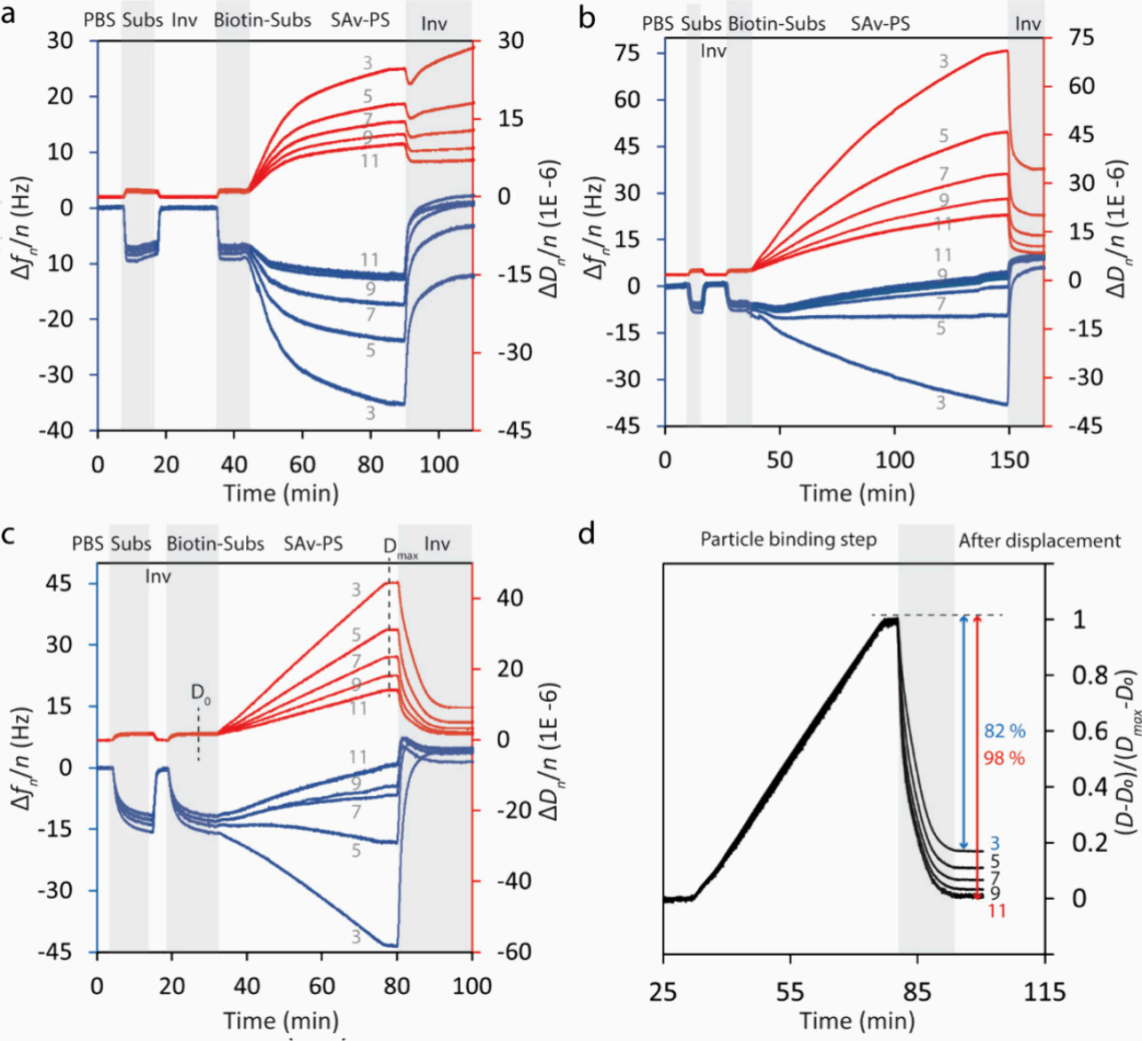
QCM-D monitoring of the particle dissociation by DNA displacement from surfaces coated with PLL (a), PLL/PAA (b), and thiol SAM (c). The blue and red curves show the frequency and dissipation changes, respectively, and the integers represent the harmonic numbers. The particle displacement assay was performed after the dynamicity of the DNA receptor actuator was checked, as can be seen in the first and second steps of the QCM-D curves. (d) Normalized dissipation responses (*D* – *D*_0_)/(*D*_max_ – *D*_0_) for the SAM-coated surface (data from panel c).

Figure 3 shows the particle displacement assay on different modified surfaces. Figure 3a shows the particle displacement assay on a PLL-OEG_4_-Tz-coated surface. The incumbent-immobilized surface was first hybridized with the substrate and then displaced by adding the invader to check the efficiency of the DNA actuator. The surface was then hybridized again with the biotin-labeled substrate (biotin-subs) to create a layer of biotin-labeled dsDNA and subsequently flushed with the SAv-coated PS particle solution. A significant increase in the dissipation response and marked variations in the frequency and dissipation responses for different harmonics are characteristic for the deposition of microparticles on the surface. The addition of the invader strand led to dissociation of the specifically anchored particles from the surface. Although a considerable restoration in frequency shift was observed, the dissipation change remained almost unaffected upon the invader addition. The positive frequency shift upon the addition of the invader can be explained as a consequence of mass decrease upon displacement of DNA strands in regions between the particles and also dissociation of a small portion of particles that were specifically anchored. However, restoration in Δ*f* can be misleading because the incomplete restoration of Δ*D* can instead be inferred as the continued presence of microparticles on the surface.

The incomplete particle dissociation on the PLL-coated surface can be explained as a consequence of the partial nonspecific electrostatic adsorption of negatively charged particles on the surface because it possesses an overall positive charge from the free amine groups of the PLL. Although the antifouling property of the PLL-OEG_4_ chemistry can potentially be improved by increasing the grafting density of OEG_4_ moieties in the structure of the polymer, there is a limitation to increasing the percentage of these antifouling agents. A minimum degree of free amine groups (about 60%) is required to guarantee stable adsorption of the polymer to the surface.^21^ Hence, to evaluate the effects of electrostatic adsorption and also improve the particle dissociation in PLL chemistry, a polyanionic polymer, PAA, was used to suppress the surface charge of PLL.

Coating of the PAA polymer was performed after the TCO-DNA immobilization step to prevent the repulsive effect of PAA on the TCO and Tz reaction. The resulting surface presents a similar ssDNA and, consequently, biotin-labeled dsDNA density (Figure S2). Figure 3b shows the frequency and dissipation time traces of the particle binding and dissociation assay on the PLL/PAA-coated surface. Similar to the PLL-coated surface, a significant increase in the dissipation response, as well as increased spread in the frequency and dissipation responses, indicate the deposition of microparticles on the surface. However, both frequency and dissipation responses show a significantly better restoration than the PLL-coated surface after the addition of the invader. This improved restoration in Δ*D* can be explained as the result of the suppressed electrostatic force on the PAA-coated surface due to the overall negative surface charge after PAA adsorption. Although suppressing the electrostatic force by PAA can improve the antifouling property of the surface and the particle dissociation, other adhesive forces such as van der Waals forces^14^ may still play a role as another origin of nonspecific binding of protein-coated microparticles on the PLL/PAA-coated surface (Figure 3b).

Performing the displacement assay on an MCH-coated surface showed an improved restoration in Δ*D* for different individual harmonics in comparison to, in particular, the PLL and also the PLL/PAA surfaces (Figure 3d). This improved particle dissociation may be explained as the result of a different, possibly more upright,^23^ presentation of dsDNA on the hydroxy-terminated SAM-coated surface, which would facilitate particle dissociation upon DNA displacement.

### Stiffness of Contact: A Signature of Binding Specificity?

As shown in Figure 3, Δ*f* in the particle displacement step can be misleading for analyzing particle dissociation. For the PLL-coated surface (without additional PAA adsorption), the dissipation signal remains nearly unchanged after adding the invader, while the frequency change represents a very good restoration for some of the harmonics. For SAM and PLL/PAA-coated surfaces, Δ*f* shows a restoration step that is larger than the original shift observed upon particle binding, while restoration of the related dissipation response remains incomplete. Evaluating the degree of particle dissociation using Δ*D* restoration is not straightforward either. Although the restoration of Δ*D* in individual harmonics can give insight into the particle dissociation efficiency, normalizing the dissipation change harmonics by (*D* – *D*_0_)/(*D*_max_ – *D*_0_) shows different restoration values for different harmonics (82–98%, Figure 3d).

We employ the coupled-resonator model^20^ to explain this behavior. Microparticles binding to the surface of an oscillator behave as coupled resonators.^20^ The spring constant describing the interactions between the microparticle and the oscillating crystal affects the overall frequency response of the system. Strongly bound particles follow the inertial loading theory in which the deposited mass leads to a negative frequency response. In contrast, in a weaker particle–surface contact, the frequency change increases upon particle landing, so-called elastic loading.^33^ The latter is a consequence of the microparticle’s inertia, such that during oscillations the particle has insufficient time to respond and a restoring force is exerted on the quartz crystal.^34^ Hence, depending on the stiffness of the contact and the mass of the particles, particle landing may cause positive or negative frequency shifts in a given harmonic.^35^ This model helps explaining particle binding and dissociation on differently modified surfaces. As can be seen in Figure 3a–c, although the dissipation responses increase upon particle binding, dissimilar frequency responses appear for different surfaces. Both positive (for large overtones) and negative (for lower overtones) frequency shifts can be observed in the frequency harmonics of the SAM and the PLL/PAA-coated surface during the particle binding steps, whereas the PLL-coated surface shows only negative shifts. The overall negative Δ*f* for the PLL-coated surface can be explained as a consequence of the strong binding of negatively charged particles on a positively charged, PLL-coated oscillator. By suppressing the electrostatic force in the PLL/PAA-coated surface, the particles become more free to vibrate with respect to the surface, which leads to a positive shift in Δ*f*. This is accompanied by a better restoration in Δ*D* during invader addition. Similar behavior can be observed for the SAM-modified surface, in good agreement with the improved particle dissociation in the invader addition step (Figure 3c). It is important to consider that the density of dsDNA in the particle interface is almost similar for the modified surfaces (first steps in frequency responses in Figure 3a–c).

**Figure 4:**
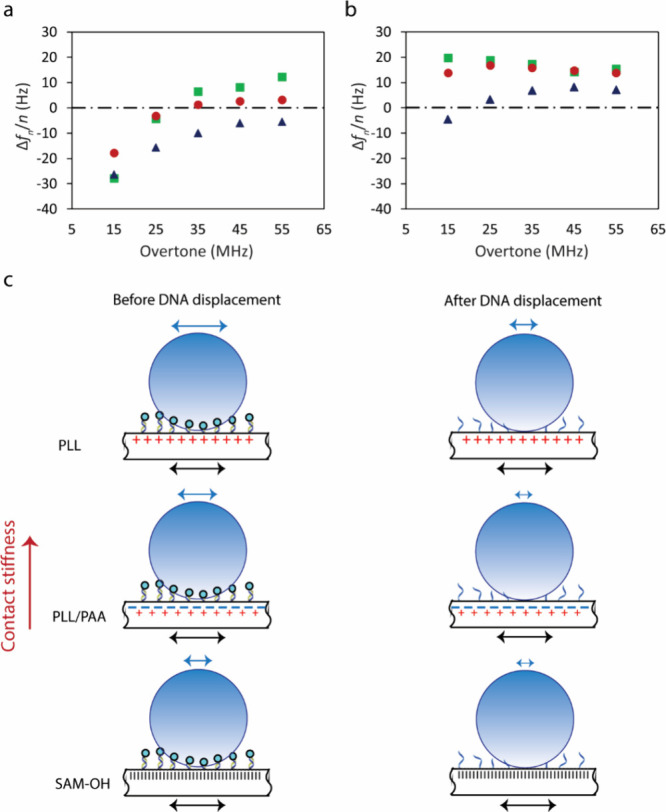
Frequency shifts versus the harmonic number representing *f*_ZC_ in SAM-coated (green ■), PLL/PAA-coated (red ●), and PLL-coated (blue ▲) surfaces, both before (a) and after (b) adding the invader. The values shown here were extracted from the frequency harmonics 45 min after flushing the surface with the particle solution so that surfaces with a similar amount of biotin-labeled dsDNA receptors present similar dissipation changes. (c) Schematic representation of particle oscillation on the PLL, PLL/PAA, and hydroxy-terminated SAM-coated surfaces before and after DNA displacement. Particles bind to the surfaces in a multivalent manner. DNA displacement gradually decreases the stiffness of contact between particles and the surface. This causes the particle motion to stop following the surface and exert a restoring force on the surface. Blue and black arrows show the relative magnitude of oscillation of a particle and the surface, respectively.

The nature of particle binding can be shown by plotting the frequency shift versus overtone number (Figure S3) to obtain *f*_ZC_, i.e., the frequency at which Δ*f* switches sign from negative at *f* < *f*_ZC_ to positive at higher harmonics. This quantity characterizes contact stiffness in the coupled-resonator model.^33^Figure 4a shows that *f*_ZC_ exhibits a monotonic relationship with the particle dissociation efficiency. In the PLL/PAA-coated surface (red), *f*_ZC_ appears at lower frequencies in comparison to the PLL-coated surface (blue). *f*_ZC_ for the SAM-coated surface (green) shows an even smaller value than the PLL/PAA-coated surface, in good agreement with the improved particle dissociation from this surface. Because surfaces with smaller *f*_ZC_ show improved particle dissociation, we can consider *f*_ZC_ as a parameter of binding specificity at a given density of receptors. Hence, relating *f*_ZC_ in the particle binding step to Δ*D* in the particle displacement step can be considered to be a mutually consistent test for the specificity of particle binding on receptor-modified surfaces.

The same approach can be used to explain the behavior of the nonspecifically adsorbed particles after addition of the invader strand (Figure 4b). This step depletes the biotin-labeled dsDNA at the interface (also outside the particle contact area) and weakens the contact stiffness of nonspecifically adsorbed particles.^34^ The decreased stiffness of contact can be deduced from Figure 4b and is illustrated schematically in Figure 4c. For the PLL-coated surface, nonspecifically adsorbed particles (Figure 4b, blue) present lower contact stiffness (*f*_ZC_ has shifted toward lower frequencies)^34^ in comparison to the condition before DNA displacement (Figure 4a, blue), which can be due to the effect of diminished multivalency. The PLL/PAA- and SAM-coated surfaces show similar behavior: the nonspecifically adsorbed particles possess less stiffness after the addition of the invader. This leads to Δ*f* values that are independent of the harmonic number and such a small value of *f*_ZC_ that it lies below the first overtone and thus cannot be observed (Figure 4b, red and green, respectively). Hence, the nonspecifically adsorbed particles on the PLL/PAA and SAM surfaces behave differently from those on the PLL surface, which may originate from other forces such as van der Waals and hydrophobic interactions. Hence, analyzing the behavior of particles after DNA displacement helps to reveal the nature of nonspecific binding.

Our analysis indicates that particles show different behavior before and after DNA displacement. Before displacement, the *f*_ZC_ is a consequence of populations of both specifically and nonspecifically bound particles that are anchored multivalently. After displacement, *f*_ZC_ is determined solely by nonspecifically adsorbed particles on the semipristine surface, and the effect of multivalency is diminished. Implementing a model for the quantification of the fraction of dissociated particles by Δ*D* restoration would not be straightforward because two inhomogeneous populations of particles must be described simultaneously. This is further evidenced by noting that, in the coupled-resonator models,^33,34,36,37^*f*_ZC_ also corresponds to the maximum dissipation change. This is not observed in the particle binding steps in Figure 3a–c. We also note that recent calculations by Fouxon et al.^38^ indicate a strong influence of hydrodynamics on the oscillator–surface interactions, which may need to be taken into account in a quantitatively accurate model. Because these calculations have not been extended to the case of tethered particles, however, we cannot directly compare them to our results here.

### Effect of the dsDNA Density on Particle Displacement

**Figure 5:**
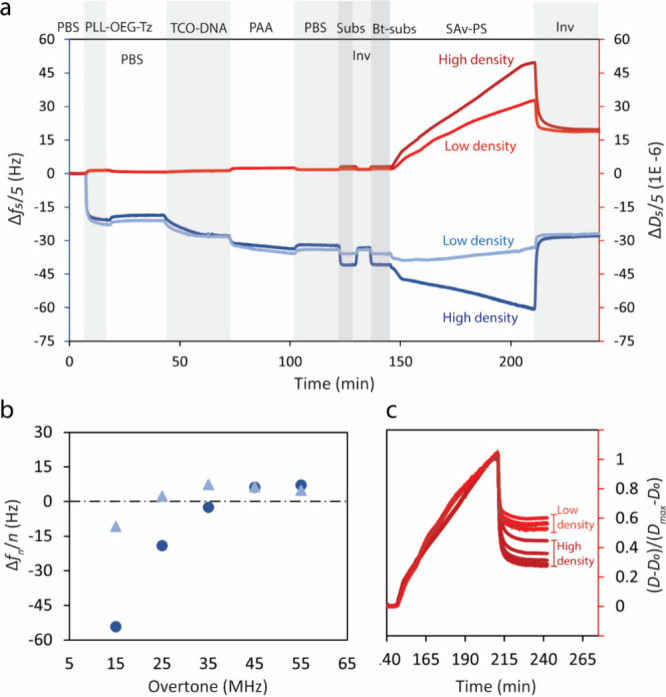
(a) Frequency and dissipation time traces of the particle displacement for different primary duplex densities (dark blue and dark red, high density; sky blue and red, low density). (b) Frequency shifts versus overtone numbers. *f*_ZC_ is lower for a lower probe density (light blue). (c) Normalized dissipation responses (*D* – *D*_0_)/(*D*_max_ – *D*_0_) for high- and low-density surfaces.

Microparticles bind to the dsDNA-coated surface in a multivalent manner. However, this is not favorable in a particle-based DNA sensor because multiple target DNAs are required to displace a specifically anchored particle. The ideal condition would be a DNA probe density where particles are bound to the surface by only a single dsDNA bridge. Releasing such particles by strand displacement promises to achieve DNA detection at a single-molecule level. On the other hand, decreasing the density of dsDNA may increase nonspecific adsorption of negatively charged particles. As concluded from the PAA-coated surface experiment, inverting the surface charge can improve the dissociation of negatively charged particles. The presence of dsDNA on the SAM-coated surface can also be a reason for the improved particle displacement. Hence, it should be possible to improve the particle binding specificity by increasing the density of dsDNA using a neutral sequence that acts as an additional spacer. At higher densities of dsDNA, particles may get distanced more strongly from the surface, which would facilitate the dissociation in the displacement step. To analyze the effect of the dsDNA density on particle displacement, the same PLL-OEG_4_-Tz polymer was used to generate two different densities of DNA receptors. To generate the surface with higher density, the PLL-coated surface was flushed with 1 μM incumbent DNA solution (similar to the procedure in Figures 2 and 3). For the lower density, a solution with 200 nM incumbent DNA, and 800 nM poly(A) ssDNA of the same length was employed. As shown in Figure 5a, the frequency responses in high- and low-density samples show a similar negative shift when the TCO-DNA solution is introduced, indicating equal functionalization for the TCO-DNA samples on a PLL-OEG_4_-Tz-coated surface with a given degree of Tz functional groups. After TCO-DNA was immobilized, the surfaces were coated with PAA and then hybridized with substrate DNA. As can be seen in the substrate hybridization step, Δ*f* is about 5 times larger for the high-density surface, representing different densities of the incumbent strand and, consequently, a biotin-labeled primary duplex on the surface.

As can be seen in the particle displacement step, the surface with a higher density of the primary duplex shows ameliorated particle displacement. This can be due to the increased negative charge and also the increased repulsion between particle and surface due to the DNA adopting a more extended configuration at a higher density of dsDNA. However, as can be seen in Figure 5b, the stiffness of the contact is increased at higher density, as reflected by the larger *f*_ZC_. This behavior, however, contradicts the monotonic relationship between smaller *f*_ZC_ and increased particle dissociation observed in Figure 4. Using *f*_ZC_ as a signature of binding specificity may only be valid for surfaces with an almost equal density of receptors. This is because increasing the multivalency increases the stiffness of contact, causing *f*_ZC_ to shift toward higher frequencies.

## Conclusion

We explored the use of multiharmonic QCM-D for deciphering the binding specificity of microparticles on various modified surfaces, utilizing TMSD as a dynamic actuator in a microparticle-based displacement assay. We showed that, although PLL chemistry with a high degree of OEG_4_ moieties exhibits ideal antifouling properties for surface-tethered DNA, nonspecific adsorption of negatively charged microparticles can still impede particle dissociation upon DNA displacement. We showed that suppressing the electrostatic forces by an anionic polymer, PAA, can improve particle dissociation from the PLL-coated surface. However, the results showed that other adhesive forces still play a role and hamper particle dissociation in the PLL/PAA-coated surface. The highest particle dissociation was observed for the hydroxy-terminated SAM-coated surface.

We employed *f*_ZC_ to explain the nature of particle binding and validate the restoration of Δ*D* in the particle dissociation step. We observed that, for a given density of dsDNA, surfaces with a smaller *f*_ZC_ showed improved particle dissociation, leading to the conclusion that *f*_ZC_ can be employed as a signature of binding specificity. Hence, *f*_ZC_ in the particle binding step and Δ*D* in the displacement step can be regarded as synergic measures for assessing the specificity of particle binding on dsDNA-modified surfaces.

## Supporting Information Available

Analysis of the DNA density in the contact area between the particle and substrate, QCM data for layers with an additional PAA coating, QCM data and procedure on how to extract *f*_ZC_, and optical microscopy image of bound particles (PDF)
