# Supplementary material for: Analyzing Binding Specificity in a Microparticle-Based DNA Displacement Assay Using Multiharmonic QCM-D
Source: Langmuir. 2024 Nov 25;40(49):26067–75. doi: 10.1021/acs.langmuir.4c03510 (PMC11636201; doi:10.1021/acs.langmuir.4c03510)
Supplement: Supplementary file 1 — la4c03510_si_001.pdf [file la4c03510_si_001.pdf]

# SUPPORTING INFORMATION

# Analyzing binding specificity in a microparticle-based DNA displacement assay using multi-harmonic QCM-D

*Taghi Moazzenzade, Luna Loohuis, Serge Lemay,* Jurriaan Huskens**

MESA+ Institute and Faculty of Science & Technology, University of Twente, P.O. Box 217, 7500 AE, Enschede, The Netherlands.

**CONTENT**

A) Calculating the amount of DNA in the particle-surface gap……………………………...………….. S2

B) Coating the DNA-coated surface with PAA polymer………………………..…………………. S3

C) Extracting $f_{zc}$ from multi-harmonic QCM-D measurements……………………………...…….. S4

D) Optical microscopy image of particles on a dsDNA-coated surface ……………………………… S5

### *A) Calculating the amount of DNA in the particle-surface gap*

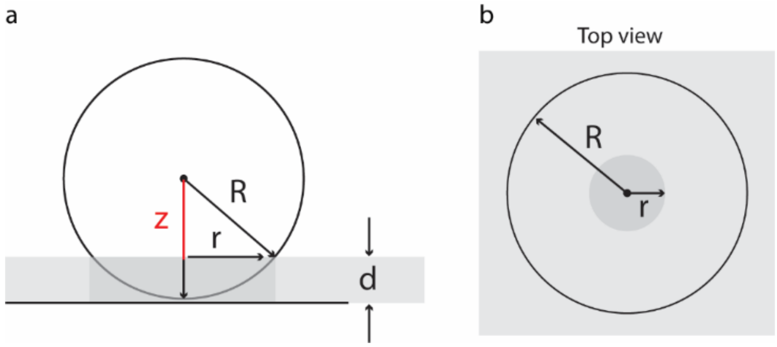


**Figure S1.** (a) and (b), Schematic representations of the contact area between the SAv-PS with a diameter of 400 nm and the dsDNA-coated surface. The contour length ($d$) of the B-DNA[30] for the primary duplex (11 bp) is considered ≈ 3.5 nm. Hence, the diameter ($r$) of the contact area between the particle and the dsDNA brush can be calculated by

$$z = R - d(R-d)^2 + r^2 = R^2$$

$$\Rightarrow r = \sqrt{d\,(2R-d)}$$

$$r \approx \sqrt{2dR} \text{ when } d << R$$

$$r \approx \sqrt{2 \times 3.5 \times 400} \approx 53 \text{ nm}$$

The contact area between the particle and the surface (dark grey) can be obtained by$A = \pi r^2 \approx$ 8800 $nm^2$

Considering the ideal stickiness of the surface due to a high density of biotin-dsDNA, ≈ 4 dsDNA per $(10\ nm)^2$ obtained from the Sauerbrey equation (Figure 2b, step 3), and the average density of 1 streptavidin per $(10\ nm)^2$ on the particle surface, the number of contacts by dsDNA between the surface and a microparticle is ≈ 88. However, this is a very rough estimate. The average number of contacts can be more because an immobilized streptavidin can bind to maximum two biotin-

labeled dsDNA. Steric hinderance can however prevent every possible pair from binding, reducing the number of bonds.

### *B) Coating the DNA-coated surface with PAA polymer*

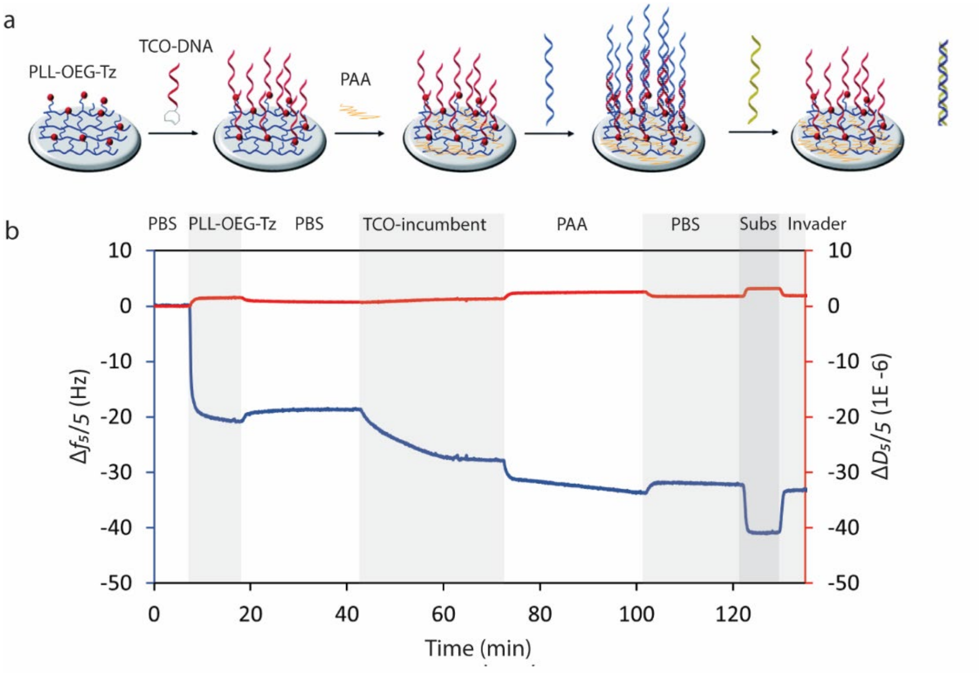


**Figure S2.** Schematic representation (a), and the QCM-D time traces (b) for the coating of PAA on a PLL-coated surface. Coating of the PAA polymer was performed after the TCO-DNA immobilization step to prevent the repulsive effect of PAA on the TCO and Tz reaction. The scheme is adapted from ref. 20.

### *C) Extracting $f_{zc}$ from multi-harmonic QCM-D measurements*

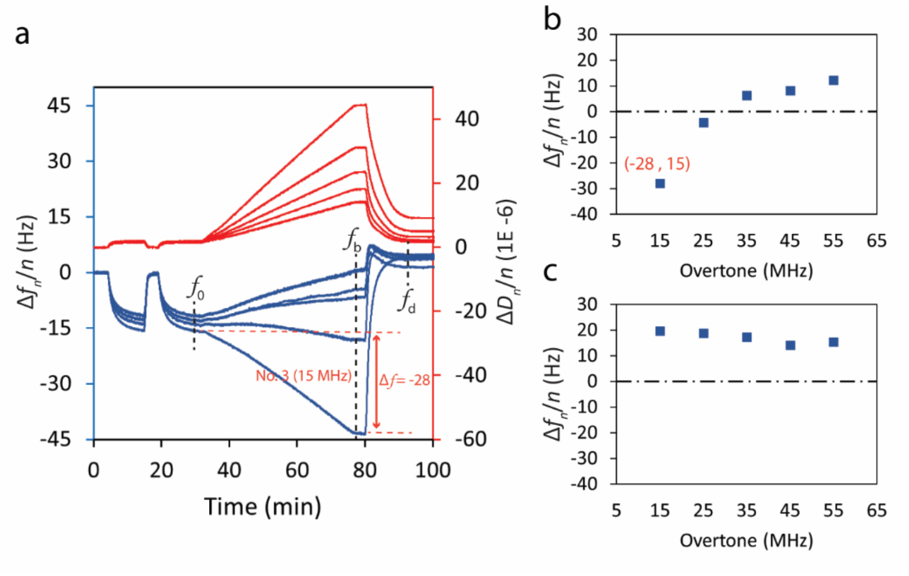


**Figure S3.** Extracting $f_{zc}$ from multi-harmonic QCM-D measurements. Measurement over the course of the particle displacement assay (a). $\Delta f$ versus harmonics following the particle binding step (b), and after DNA displacement (c).

### *D) Optical microscopy image of particles on a dsDNA-coated surface*

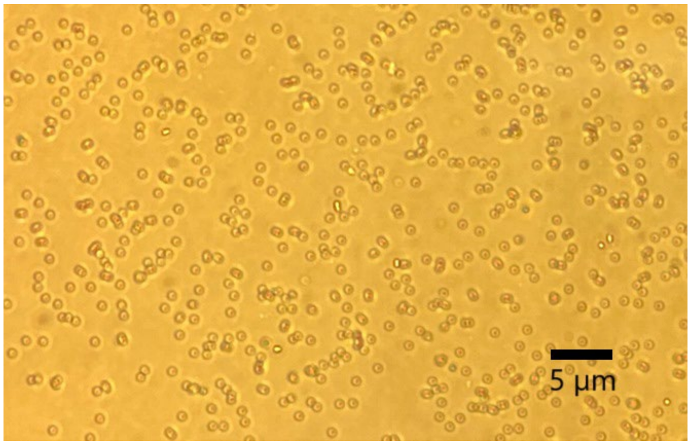


**Figure S4.** Optical microscopy image showing 800 nm particles on a dsDNA-coated surface prior to DNA displacement. The observed density may be lower than the actual density in QCM-D, as some particles could dissociate during the disassembly of the chip for optical microscopy analysis.
